# Effects of respiratory sarcopenia on the postoperative course in elderly lung cancer patient: a retrospective study

**DOI:** 10.1186/s13019-024-03185-w

**Published:** 2025-01-18

**Authors:** Dong Jae Han, Kwon Joong Na, Taeyoung Yun, Ji Hyeon Park, Bubse Na, Samina Park, Hyun Joo Lee, In Kyu Park, Chang Hyun Kang, Young Tae Kim

**Affiliations:** 1 Department of Thoracic and Cardiovascular Surgery, Seoul National University Hospital, 101 Daehak-Ro, Jongno-Gu, Seoul, 03080 Republic of Korea; 2 Department of Thoracic and Cardiovascular Surgery, Seoul National University College of Medicine, Seoul, Republic of Korea; 3 Seoul National University Cancer Research Institute, Seoul, Republic of Korea

**Keywords:** Lung cancer, Respiratory sarcopenia, Postoperative outcome

## Abstract

**Objectives:**

Recently, sarcopenia has been linked to unfavorable outcomes in various surgical procedures, including lung cancer surgery. This study aimed to investigate the impact of respiratory sarcopenia (RS) on postoperative and long-term outcomes in elderly patients undergoing lung cancer surgery.

**Methods:**

This retrospective study included patients aged 70 years and older who underwent lobectomy with curative intent for lung cancer between 2017 and 2019. RS was defined as having values below the median for both the L3 skeletal muscle index, measured from preoperative PET-CT images, and peak expiratory flow (PEF). An inverse probability of treatment weighting (IPTW) approach was applied to balance covariates between the RS and non-RS groups. Baseline characteristics and postoperative outcomes were compared between groups using t-tests and chi-square tests. Kaplan–Meier curves and log-rank tests were used to compare overall and recurrence-free survival. Multivariable logistic regression analysis incorporating IPTW weights was performed to assess the impact of RS on respiratory complications.

**Results:**

A total of 509 patients were included, of whom 123 (24.2%) had RS. After IPTW adjustment, baseline characteristics, including pulmonary function, were similar between the RS and non-RS groups. All patients underwent lobectomy, with 78.8% of the RS group and 80.9% of the non-RS group undergoing minimally invasive surgery. The RS group had a significantly higher rate of respiratory complications compared to the non-RS group (14.5% vs. 7.7%, *p* = 0.041). Multivariable logistic regression analysis showed that male sex (odds ratio = 15.2, *p* < 0.01) and lower D_LCO_ (odds ratio = 0.96, *p* < 0.01) were significantly associated with respiratory complications, whereas RS did not show a significant association (*p* = 0.05). No significant differences were found in overall survival (*p* = 0.11) or recurrence-free survival (*p* = 0.51) between the groups.

**Conclusions:**

In this study, RS had a limited impact on both postoperative and long-term outcomes in elderly patients undergoing lung cancer surgery. These findings suggest that other factors, such as D_LCO_ and male sex, may play a more prominent role in predicting respiratory complications.

## Background

Surgical resection is the cornerstone of treatment for early-stage lung cancer, with several factors—including age, operative procedure, pulmonary function, and lung cancer stage—proven to be prognostic indicators of both short- and long-term outcomes [1–3]. More recently, a patient’s functional status has also been considered a significant determinant of outcomes in patients with lung cancer. Notably, many clinicians have shown interest in sarcopenia, a syndrome highly prevalent in the elderly population [4–7]. The prevalence of sarcopenia tends to increase with advancing age, with a steep rise observed between 65 and 75 years [7, 8]. Recent studies have reported that sarcopenia affects the postoperative outcomes of gastrointestinal surgeries, such as those for esophageal and gastric cancers, as well as cardiac surgeries [9–12]. Specifically, sarcopenia has been observed to impact short- and long-term outcomes, including overall and recurrence-free survival, in lung cancer surgery [13, 14].

Recent studies have demonstrated that respiratory sarcopenia (RS), defined as inspiratory muscle weakness, is associated with postoperative pulmonary complications [15]. Effective inspiration is crucial for preventing fatal pulmonary complications in patients who have undergone lung cancer surgery. Assessing coughing ability through the evaluation of respiratory muscle strength and expectoration power may be particularly beneficial, especially in elderly patients [16, 17]. Currently, the majority of lung surgeries are performed using minimally invasive surgery (MIS) and managed postoperatively with an enhanced recovery after surgery (ERAS) protocol. The adoption of MIS and ERAS has led to a significant reduction in the rate of pulmonary complications, even in elderly patients [18–21]. Though the clinical significance of RS in elderly patients has been demonstrated in previous studies, whether it affects the surgical outcomes of lung cancer has not been studied yet.

Therefore, this study aimed to investigate the effects of RS on the early and long-term outcomes of elderly patients with lung cancer undergoing surgical resection.

## Methods

### Ethical statement

This study was reviewed and approved by the institutional review board of Seoul National University Hospital (2022-02-22, H-2201-105-1291), and it followed the tenets of the Declaration of Helsinki. The requirement for informed consent of participants was waived due to the retrospective nature of the study.

### Patients

We investigated the patients who underwent curative surgery for lung cancer or suspected lung nodules between January 1, 2017 and December 31, 2019 in Seoul National University Hospital. We included patients aged 70 and older, who underwent pulmonary lobectomy with curative intent. As we utilized ^18^F-fluorodeoxyglucose positron emission tomography (PET)-computed tomography (CT) images to calculate the muscle index for sarcopenia definition, we excluded patients who did not receive preoperative PET or had PET performed at an external hospital.

### Definition of respiratory sarcopenia

Based on the definition of RS provided by Kera PT et al. [16] and Nagano et al. [17], we assessed RS by measuring whole-body muscle mass and respiratory muscle strength. We evaluated whole-body sarcopenia using the L3 skeletal muscle index (SMI) and assessed respiratory muscle strength through peak expiratory flow (PEF) from preoperative pulmonary function tests. We divided the study population into two groups using the median values of L3 SMI and PEF. Patients with both L3 SMI and PEF below the median were classified as the RS group, while those with values above the median were categorized as the non-RS group.

### Skeletal muscle index calculation

All preoperative PET-CT scans, extending from the skull base to the mid-thigh, were performed with the following parameters: section thickness of 1 mm, section interval of 1 mm, tube voltage at 100 kVp, and tube current set to 500 mA. The scans were then imported into a commercially available, deep learning–based body composition analysis software platform (version 1.0.0.0, Deep Catch, Medical IP, South Korea) [22]. This software was utilized to calculate the skeletal muscle area at the L3 vertebra level. We defined the L3 SMI as the ratio of the skeletal muscle area at L3 to the body mass index for normalization.

### Pulmonary function test

All patients underwent preoperative pulmonary function tests, which included measurements of forced vital capacity, forced expiratory volume in one second (FEV_1_), diffusing capacity of the lung for carbon monoxide (D_LCO_), and PEF. PEF represents the maximum flow attained during a forced expiration initiated at the point of maximal lung inflation. Drawing on prior research that identified correlations between PEF and sarcopenia, this study utilized PEF as a criterion to delineate RS [15, 23, 24].

### End points

The early postoperative outcomes, including the rate of all complications, respiratory complications (atelectasis requiring bronchoscopic toileting, pneumonia, reintubation for respiratory failure, a requirement for tracheostomy, or acute respiratory distress syndrome), and mortality, and the length of hospital stay, were compared between the two groups. Furthermore, we investigated whether RS influences overall and recurrence-free survival.

### Statistical analysis

Continuous variables were assessed using t-tests, while categorical variables were assessed using chi-square tests. An inverse probability of treatment weighting (IPTW) approach was used to balance covariates between the treatment groups. Propensity scores were estimated using a generalized boosted model, including age, sex, BMI, smoking status, and pulmonary function test parameters. IPTW-adjusted analyses were conducted to compare baseline characteristics and outcome variables between groups, with weighted means and standard deviations for continuous variables and weighted counts and proportions for categorical variables. Multivariable logistic regression analysis was performed to investigate the influence of RS on respiratory complications, incorporating IPTW weights. The variables included in the multivariable analysis were selected based on their known association with respiratory complications in previous studies, as well as their clinical relevance to the current research question. Specifically, age, sex, pulmonary function parameters (FEV_1_, D_LCO_), and operative approach were included, as they have been shown to impact respiratory outcomes, alongside respiratory sarcopenia, which was the primary focus of this study. Overall survival was defined as the time from the day of surgery to death from any cause. Recurrence-free survival was defined as the time from the day of surgery to the first detection of recurrence. Survival curves were depicted using the Kaplan–Meier method, and a log-rank test was performed to evaluate statistical significance. A *p*-value < 0.05 was considered statistically significant. All statistical analyses were performed using R version 4.0.0 (R Foundation for Statistical Computing, Vienna, Austria).

## Results

### Baseline characteristics the study population

In this study, 509 patients were enrolled, with a mean age of 75.3 ± 4.0 years and 308 (60.5%) males. The median (interquartile range) L3 SMI values were 537.6 (497.4–580.3) and 384.5 (354.3–422.1) for males and females, respectively. The median (interquartile range) PEF values were 106.0% (92.0–120.0%) and 114.0% (99.5–128.0%) for males and females, respectively. Patients with L3 SMI and PEF values below the median for their respective sex were placed in the RS group (n = 123, 24.2%), while the others were categorized as the non-RS group (n = 386, 75.8%) for subsequent analysis.

The mean age of the RS group was higher than in the non-RS group (76.2 ± 4.3 vs. 75.0 ± 3.9 years, *p* = 0.005). No significant differences were found in other clinical variables, including body mass index, smoking history, and preoperative clinical stage. Pulmonary function test values were significantly lower in the RS group compared to the non-RS group (*p* < 0.05 for all PFT values, Table 1). After applying IPTW adjustment, the baseline characteristics, including pulmonary function and other clinical variables, were well balanced between the RS and non-RS groups (Table 1).

**Table 1:** Preoperative characteristics of the patients

Variable	Unadjusted comparison			IPTW-adjusted comparison			
	RS Group (n = 386)	non-RS Group (n = 123)	*P*	RS Group	non-RS Group	*P*	SMD
Age (year)	75.02 ± 3.89	76.17 ± 4.26	0.005	75.44 ± 4.26	75.12 ± 3.94	0.521	0.078
Sex, male, n (%)	232 (60.1)	76 (61.8)	0.752	55.2%	60.8%	0.351	0.113
Body Mass Index (kg/m^2^)	22.66 ± 3.07	23.93 ± 3.27	0.413	23.51 ± 3.14	23.65 ± 3.10	0.685	0.046
L3 SMI	492.85 ± 99.55	429.06 ± 80.52	< 0.001				
Ever-smoker, n (%)	211 (54.7)	70 (56.8)	0.678	51.7%	55.4%	0.528	0.076
Pulmonary function test (%)							
FVC (liter)	3.14 ± 0.72	2.84 ± 0.72	< 0.001	2.88 ± 0.69	3.13 ± 0.72	0.001	0.362
FVC (% predicted)	104.97 ± 15.19	96.20 ± 16.33	< 0.001	104.69 ± 22.24	109.04 ± 21.59	0.130	0.199
FEV_1_ (liter)	2.20 ± 0.46	1.88 ± 0.42	< 0.001	1.96 ± 0.41	2.67 ± 8.79	0.148	0.114
FEV_1_ (% predicted)	111.13 ± 21.66	97.32 ± 19.41	< 0.001	104.69 ± 22.24	109.04 ± 21.59	0.130	0.199
D_LCO_ (mL/mmHg/min)	14.70 ± 3.22	13.54 ± 2.90	< 0.001	13.57 ± 2.84	14.66 ± 3.18	0.001	0.361
D_LCO_ (% predicted)	95.35 ± 20.14	91.20 ± 16.77	0.039	92.80 ± 17.54	94.85 ± 19.94	0.354	0.109
PEF (liter/min)	7.02 ± 1.71	5.43 ± 1.18	< 0.001				
PEF (% predicted)	115.11 ± 23.12	90.67 ± 15.40	< 0.001				
Preoperative Stage							
Clinical T stage			0.152			0.786	0.114
T1	174 (45.1)	44 (35.8)		42.2%	44.5%		
T2	161 (41.7)	58 (47.2)		42.7%	41.9%		
T3	43 (11.1)	15 (12.2)		11.0%	11.3%		
T4	8 (2.1)	6 (4.9)		4.2%	2.3%		
Clinical N stage			0.945			0.873	0.093
N0	342 (88.6)	108 (87.8)		89.0%	88.7%		
N1	33 (8.5)	10 (8.1)		7.5%	8.2%		
N2	10 (2.6)	5 (4.1)		3.5%	3.1%		
Neoadjuvant therapy, n (%)	3 (0.8)	1 (0.8)		1.4%	0.7%		

SMI = skeletal muscle index; FVC = Forced vital capacity; FEV_1_ = forced expiratory volume in one second; D_LCO_ = diffusing capacity of carbon monoxide; PEF = Peak expiratory flow

### Operative findings and early postoperative outcomes

The operative findings and early postoperative outcomes were compared using IPTW adjustment (Table 2). All patients underwent lobectomy, with minimally invasive surgery (MIS) performed in 78.8 and 80.9% of cases in the RS and non-RS groups, respectively, showing no significant difference between the groups (*p* = 0.633). The 30-day mortality rate was similar between the RS and non-RS groups (0.2% vs. 0.9%, *p* = 0.303), and the 90-day mortality rate also showed no statistically significant difference (0.5% vs. 2.0%, *p* = 0.087). The overall complication rate did not significantly differ between groups (30.3% vs. 27.8%, *p* = 0.630). However, the RS group had a significantly higher rate of respiratory complications compared to the non-RS group (14.5% vs. 7.7%, *p* = 0.041). There were no significant differences in the pathological T stage (*p* = 0.728) or N stage (*p* = 0.906) between the groups.

**Table 2:** IPTW-adjusted analysis of operative details and postoperative results

Variables	RS Group (%)	non-RS Group (%)	*P*
*Approach*			
MIS (%)	78.8	80.9	0.633
30-day mortality (%)	0.2	0.9	0.303
90-day mortality (%)	0.5	2.0	0.087
All complications (%)	30.3	27.8	0.630
Respiratory complications (%)	14.5	7.7	0.041
*Pathological T stage*			0.728
T1	33.0	28.2	
T2	50.8	54.8	
T3	11.4	10.2	
T4	4.9	6.8	
*Pathological N stage*			0.906
N0	75.4	75.1	
N1	11.1	9.9	
N2	13.5	15.0	

RS = respiratory sarcopenia; MIS = minimally invasive surgery

To assess the impact of RS on respiratory complications while adjusting for other factors, a multivariable logistic regression analysis was performed, including age, sex, pulmonary function test parameters (FEV_1_, D_LCO_), and the operative approach. The analysis revealed that respiratory complications were significantly associated with male sex (odds ratio = 15.2, 95% confidence interval: 4.08–56.76, *p* < 0.01) and lower D_LCO_ (odds ratio = 0.96, 95% confidence interval: 0.94–0.97, *p* < 0.01). Respiratory sarcopenia showed a marginal effect on respiratory complications (odds ratio = 0.44, 95% confidence interval: 0.20–1.00, *p* = 0.05), but it was not statistically significant. There was no significant association between FEV_1_ or MIS and respiratory complications. (Table 3).

**Table 3:** Multivariable logistic regression analysis for respiratory complications (IPTW-adjusted

Variables	*p*-value	OR (95% CI)
Age	0.62	1.02 (0.93–1.13)
Male sex	< 0.01	15.2 (4.08–56.76)
Respiratory sarcopenia	0.05	0.44 (0.20–1.00)
Pulmonary function test		
FEV_1_	0.69	1.00 (0.98–1.03)
D_LCO_	< 0.01	0.96 (0.94–0.97)
MIS	0.27	1.50 (0.73–3.07)

CI = Confidence interval; OR = odds ratio; FEV_1_ = forced expiratory volume in one second; D_LCO_ = diffusing capacity of carbon monoxide; MIS = minimally invasive surgery

### Impact of respiratory sarcopenia on long-term outcomes

**Fig. 1:**
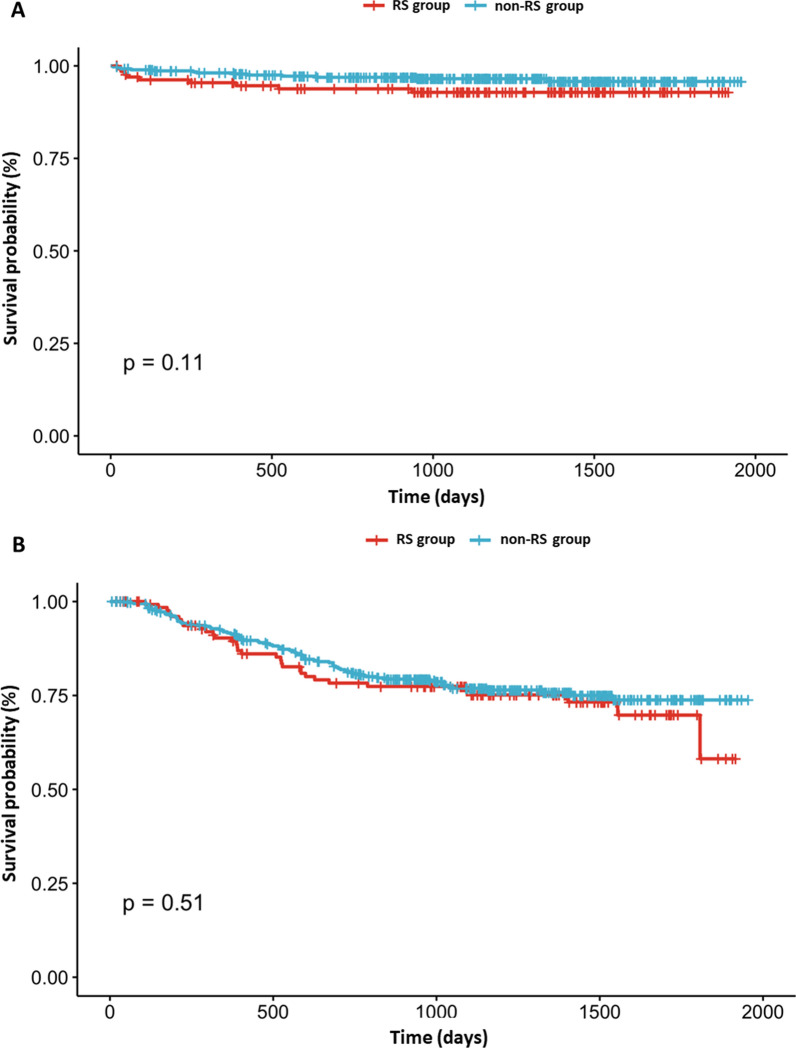
Overall survival rate (**A**) and recurrence-survival rate (**B**) of all study population. Survival was not different between two groups

The Kaplan–Meier method depicted the overall (Fig. 1A) and recurrence-free (Fig. 1B) survival for both the RS and non-RS groups. Median follow-up duration of all study cohort was 38.6 months (interquartile range, 28.8–49.5 months). There was no significant difference in long-term survival between groups (*p* = 0.11 for overall survival and *p* = 0.51 for recurrence-free survival).

## Discussion

This study aimed to investigate the influence of RS on postoperative outcomes in patients aged 70 or older who underwent lobectomy with curative intent for lung cancer. No differences in postoperative outcomes were observed between the RS and non-RS groups, except for a significantly higher incidence of respiratory complications in the RS group. Multivariable analysis revealed that respiratory complications were associated not with RS, but with male sex and low D_LCO_. Furthermore, the study demonstrated that RS had no association with long-term outcomes.

As the global population ages, the clinical significance of sarcopenia has gained more attention, highlighting its impact on the general population and associated issues [4–8]. Recent studies have increasingly shown that sarcopenia affects both the early and long-term outcomes of surgical patients [9–12]. Additionally, RS, a specific type of sarcopenia related to respiratory muscle strength, has attracted attention in patients with lung cancer due to its importance in predicting and preventing pulmonary complications, which is more critical than in other solid tumors [13–17]. The pathophysiology of sarcopenia is complex, and there is no universal consensus on the definition of RS. In this study, we defined RS using L3 SMI and PEF. International guidelines on sarcopenia [4, 5, 25] suggest that L3 skeletal muscle mass, measured by CT, is a reliable indicator for evaluating muscle mass. There are various methods to estimate respiratory muscle strength, with PEF recognized as a robust indicator [15–17, 23, 24]. Since patients with lung cancer undergoing surgical resection routinely undergo PET-CT and PFT as part of their preoperative workup, we believe that measuring L3 SMI and PEF is a practical approach in clinical practice.

Sun et al. [15] recently defined RS using thoracic muscle mass and PEF, demonstrating that RS is a significant prognostic factor for patients with lung cancer. Their study was among the first to provide evidence supporting the identification of patients at risk of sarcopenia using the thoracic muscle index and PEF. In contrast, even though we only included elderly patients with a high prevalence of sarcopenia, our study demonstrated that RS had no discernible effect on long-term survival or immediate postoperative outcomes. RS did not show significant impact on respiratory complication in subgroup analysis by gender, though some studies have demonstrated there is some difference in clinical significance by men and women. [26]

There might be several reasons for the opposing results from the previous study. As there is no universal consensus to define RS, the results might be different from study to study. Our group defined the RS with muscle mass (L3 SMI) and power (PEF), though the definition of muscle mass is different from the study by Sun et al. [15]. Second, it should be taken into consideration that RS employed in our study is only one of the major features of the frailty syndrome. It may also be insufficient in overall reflecting the frailty of the patients. The frailty phenotype can be defined by the presence of several components besides sarcopenia, such as low physical activity, poor endurance, and weakness, that have not been evaluated in this study [27–30]. Third, there are many clinical and baseline characteristics that affects outcomes besides RS. Differing from Sun et al. [15], our study encompassed a larger cohort of patients who underwent MIS and more recent phase patients after the implementation of the ERAS protocol for lung resection surgery at our center. MIS, known for causing less injury and trauma than open surgery, has been corroborated by various studies to yield favorable postoperative outcomes and fewer complications [18, 19]. Additionally, the recent adoption of ERAS has been associated with improved postoperative outcomes for patients with lung cancer, as evidenced by a decrease in the frequency of complications and shorter hospital stays [20, 21].

### Limitation

This study has several limitations. First, its retrospective, single-center design inherently carries limitations associated with retrospective analyses. Second, we cannot entirely exclude the potential for selection bias, as patients anticipated to be intolerant to surgery might have been excluded during the preoperative work-up. Thus, the patients chosen for lung cancer surgery may consist of healthy individuals, the majority of whom do not have RS, and whose outcome is not readily affected by RS. Third, the absence of a standardized method for defining RS means that the definition used in this study may not be universally applicable.

## Conclusions

In conclusion, our study demonstrated that RS did not affect early postoperative or long-term outcomes in elderly patients who underwent curative surgical resection for lung cancer. Given that the majority of our study population underwent MIS and received ERAS, the influence of RS on the postoperative course may have been diminished.

## Data Availability

No datasets were generated or analysed during the current study.

## Abbreviations

RS: Respiratory sarcopenia
MIS: Minimally invasive surgery
ERAS: Enhanced recovery after surgery
PET-CT: Positron emission tomography-computed tomography
SMI: Skeletal muscle index
PEF: Peak expiratory flow
FEV_1_: Forced expiratory volume in one second
D_LCO_: Diffusing capacity of the lung for carbon monoxide
FVC: Forced vital capacity
CI: Confidence interval
